# Early Solid Diet Supplementation Influences the Proteomics of Rumen Epithelium in Goat Kids

**DOI:** 10.3390/biology12050684

**Published:** 2023-05-06

**Authors:** Yimin Zhuang, Xiaokang Lv, Kai Cui, Jianmin Chai, Naifeng Zhang

**Affiliations:** 1 Key Laboratory of Feed Biotechnology of the Ministry of Agriculture and Rural Affairs, Feed Research Institute, Chinese Academy of Agricultural Sciences, Beijing 100081, China; b20213040363@cau.edu.cn (Y.Z.);; 2 Guangdong Provincial Key Laboratory of Animal Molecular Design and Precise Breeding, College of Life Science and Engineering, Foshan University, Foshan 528225, China; 3 Division of Agriculture, Department of Animal Science, University of Arkansas, Fayetteville, AR 72701, USA

**Keywords:** goat, rumen epithelium, proteome, solid feed, protein expression

## Abstract

**Simple Summary:**

The rumen, as a unique digestive organ of ruminants, is vitally important to their growth and production, while the rumen of young ruminants is not fully developed. Previous studies have proven that early solid diet supplementation can significantly promote the development of rumen and improve their growth performance. However, the changes in the expressed proteome and related metabolism in rumen epithelium in response to a supplemented solid diet remains unclear. In this study, we confirmed that a solid diet significantly promoted the growth performance, rumen fermentation, and rumen epithelial development of goat kids. According to proteomic analysis, we further identified the proteins and pathways related to cell growth and volatile acid metabolism which were significantly changed via solid diet supplementation. This study can support the optimal breeding strategy to improve the performance and the growth potential of young ruminants.

**Abstract:**

It is well known that solid diet supplementation in early life can significantly promote rumen development and metabolic function in young ruminants. However, the changes in the expressed proteome and related metabolism in rumen epithelium in response to a supplemented solid diet remain unclear. In this study, rumen epithelial tissue from goats in three diet regimes including milk replacer only (MRO), milk replacer supplemented concentrate (MRC), and milk replacer supplemented concentrate plus alfalfa pellets (MCA) were collected for measurement of the expression of epithelial proteins using proteomic technology (six per group). The results showed that solid diet significantly improved the growth performance of goats, enhanced the ability of rumen fermentation, and promoted the development of epithelial papilla (*p* < 0.05). Proteome analysis revealed the distinct difference in the expressed protein in the MRC and MCA group compared with the MRO group (42 upregulated proteins and 79 downregulated proteins in MRC; 38 upregulated proteins and 73 downregulated proteins in MCA). Functional analysis showed that solid diet supplementation activated a variety of molecular functions in the epithelium, including protein binding, ATP binding, structural constituent of muscle, etc., in the MRC and MCA groups. Meanwhile, the expression of proteins related to fatty acid metabolism, the PPAR signaling pathway, valine, leucine, and isoleucine degradation, and butanoate metabolism were upregulated, being stimulated by solid feed. In contrast, the proteins associated with carbohydrate digestion and absorption and glycosaminoglycan degradation were downregulated. In addition, the protein expression of enzymes involved in ketone body synthesis in the rumen was generally activated, which was caused by solid feed. In summary, solid feed promoted the development of rumen epithelium by changing the expression of proteins related to fatty acid metabolism, energy synthesis, and signal transduction. The ketone body synthesis pathway might be the most important activated pathway, and provides energy for rumen development.

## 1. Introduction

Rumen plays a key role in ruminants’ performance and production [1]. Goat kids are born with immature rumen that have no physiological or metabolic functions [2]. Physical and metabolic development of the rumen is essential for its smooth transition from non-ruminant to mature ruminant state, and for improving the growth performance of young ruminants [3]. As we know, the introduction of solid diets in early life is a vital driver for the development of rumen epithelium due to the nutrient components in solid diet [4]. Previous studies found that supplementing solid feed during the pre-weaning period can effectively increase rumen weight and papillae size, enhance the physical barrier of rumen to harmful substances, and, ultimately, have a positive effect on the health and growth of young ruminants [5,6,7]. Other studies have reported that early feeding starter can influence the gene expression of rumen epithelium in lambs and sheep, for example, downregulates genes including IL-6, IL-10, and IFN-γ [8], and upregulates genes containing MCT1, MCT4, and NHE3 [9]. Experiments with goats confirmed that high-grain diets simultaneously increased the volatile fatty acids (VFA) production and the expression of genes involved in VFA absorption and cell proliferation in rumen [10,11,12]. In calf studies, the implementation of similar methods significantly increased the expression of fat and muscle tissue synthesis genes [13]. Recently, studies on the effects of different diets on rumen epithelial mRNA gene expression have received more attention. However, few studies have investigated the expressed proteins in rumen epithelium as affected by diet. In sheep and dairy cattle, a study reported the differential expression of selected proteins related to material transport and metabolism due to changes in diet and rumen environment [14,15]. Therefore, it is necessary to perform systemic cognition on rumen epithelial protein expressions of goats that are fed a supplemented solid diet, which can help us understand the molecular mechanism of rumen development and further improve the feeding strategy for young ruminants. Isobaric tags for relative and absolute quantitation (iTRAQ) are used in quantitative proteomics due to their high sensitivity and conveniences [16]. In this study, we applied proteomic analysis based on iTRAQ [17] to evaluate changes in the protein expression of rumen epithelium in early supplementation goats compared to a control group without a solid diet. Through the exploration based on the molecular level, we learned how solid diet drives proteome changes in rumen epithelium. We hypothesized that solid feed supplementation can promote the growth of rumen epithelium via stimulating the expression of proteins related to cell development and volatile acid metabolism. This work can support the optimal breeding strategy to improve the performance and the growth potential of young ruminants.

## 2. Materials and Methods

### 2.1. Ethics Statement

The study was conducted at the Green Sheep Valley Farm in Haimen City, Jiangsu Province. Procedures for breeding and slaughtering were implemented in accordance with the Regulations for the Administration of Affairs Concerning Experimental Animals promulgated by the Ministry of Science and Technology, China, revised version, March 2017. The trial was also reviewed and approved by the Animal Ethics Committee of the Chinese Academy of Agricultural Sciences (AEC-CAAS-FRI-CAAS20180305).

### 2.2. Animals and Diets

Thirty-six pairs of twin goats with an average weight of 4.53 ± 0.52 kg were separated from their dams at 20 days of age and randomly divided into three groups. One group was fed with milk replacer only (MRO), which was provided by Beijing Precision Animal Nutrition Research Center, China, one group was fed with milk replacer supplemented with concentrate (MRC), and one group was fed with milk replacer supplemented concentrate plus alfalfa pellets (MCA). Each group had six replicates and four kids per pen as a replicate. During the trial, all goat kids had ad libitum access to water, milk replacer (MR), concentrate, and alfalfa pellets. Nutritional levels of MR, concentrate, and alfalfa pellets are shown in Table S1. At 60 days of age, six goats (healthy and BW close to the average of the corresponding groups) were chosen from each group, and slaughtered for rumen sample collection. The rumen epithelial tissue in ventral sac was quickly harvested and snap frozen in a liquid nitrogen tank for total protein extraction.

### 2.3. Determination of Rumen Fermentation Parameters and Morphology

The rumen content samples were thawed at 4 °C and then centrifuged at 2500× *g* at room temperature. Next, 1 mL of the supernatant per sample was separated and transferred into a 1.5 mL centrifuge tube which contained 0.2 mL of metaphosphoric acid solution (25% *w*/*v*). Then, the mixture was centrifuged at 10,000× *g* at 4 °C after placing in a water bath for 30 min. The collected supernatant was stored at 4 °C for the subsequent analysis. The VFA concentration was detected using gas chromatography (GC–6800, Beijing Beifen Tianpu instrument Technology, Co., Ltd., Beijing, China). The determination of enzyme activity (Pepsin, A-amylase, Lipase, Carboxymethyl Cellulase) was mainly carried out according to the operation procedure of the corresponding kits.

A 2 cm × 2 cm section of rumen epithelium tissue collected from each goat kid was directly washed with physiological saline and fixed in a 250 mL jar containing 10% neutral formalin solution after slaughter. The samples were dehydrated by different concentrations of ethanol, embedded in paraffin sections, and cut into 6 μM sections. The rumen papilla structure was observed under a light microscope at a magnification of 4 × 10 times (Olympus BX-51; Olympus Corporation, Tokyo, Japan) after staining with Yi-hong-hematoxylin (H.E.). The image-pro express image analysis processing system (Im-age-Pro Plus 6.0, Media Cybernetics, Silver Spring, MD, USA) was used to observe and measure the rumen papilla length, papilla width, lamina propria thickness, and epithelial thickness.

### 2.4. Liquid Chromatography–Tandem Mass Spectrometry (LC/MS) Analysis

Proteins were extracted by using lysis buffer 3 (Configuration method: 1. Urea 210 g, Thiourea 76 g, SDS 1 g, Tris 1.2 g, put in a beaker. 2. Add Milli-Q H2O 250 mL and put it on a magnetic agitator overnight (or completely dissolve). 3. The concentrated HCL is adjusted to pH 8.0–8.5. The volume of Milli-Q H2O is fixed to 500 mL, and then packed separately for reserve.) and two magnetic beads. The mixtures were placed into a TissueLyser for 2 min at 50 Hz to release proteins. After centrifugating, the supernatant was transferred into a new tube, and reduced with 10 mM dithiothreitol (DTT) at 56 °C for 1 h and alkylated by 55 mM iodoacetamide (IAM) in the dark at room temperature for 45 min. After centrifugation (25,000× *g*, 4 °C, 20 min), the supernatant containing proteins was quantified by Bradford assay. We mixed 15–30 μg proteins with loading buffer in centrifuge tube and heated them at 95 °C for 5 min. Then, the supernatant was centrifuged at 25,000× *g* for 5 min and loaded to sample holes in 12% polyacrylamide gel. The SDS-PAGE at a constant voltage of 120 V for 120 min was performed to detect proteins quality. Once finished, we stained the gel with Coomassie Blue for 2 h, then added destaining solution (40% ethanol and 10% acetic acid) and placed it on a shaker (exchange destaining solution 3~5 times, 30 min a time). The protein solution (100 μg) with 8 M urea was diluted 4 times with 100 mM Tetraethylammonium bromide (TEAB). Then, the proteins were digested at 37 °C overnight by Trypsin Gold (Promega, Madison, WI, USA) in a ratio of protein: trypsin = 40:1. After trypsin digestion, the peptides were desalted using Strata X C18 column (Phenomenex) and vacuum-dried according to the manufacturer’s protocol. The peptides were dissolved in 30 μL 0.5M TEAB with vortexing. After the iTRAQ labeling reagents were recovered to ambient temperature, they were transferred and combined with proper samples. Peptide labeling was performed by iTRAQ Reagent 8-plex Kit (8-plex iTRAQ reagent Multiplex kit, ABSciex, Framingham, MA, USA) according to the manufacturer’s protocol. The labeled peptides with different reagents were combined (Table S2), desalted with a Strata X C18 column (Phenomenex), and vacuum-dried according to the manufacturer’s protocol.

The separation of peptides was carried out on a Shimadzu LC-20AB HPLC Pump system coupled with a high pH RP column. The peptides were reconstituted with buffer A (5% ACN, 95% H_2_O, adjust pH to 9.8 with ammonia) to 2 mL and loaded onto a column containing 5 μm particles (Phenomenex). The peptides were separated at a flow rate of 1 mL/min with a gradient of 5% buffer B (5% H_2_O, 95% ACN, adjusted pH to 9.8 with ammonia) for 10 min, 5–35% buffer B for 40 min, and 35–95% buffer B for 1 min. The system was then maintained in 95% buffer B for 3 min and decreased to 5% within 1 min before equilibrating with 5% buffer B for 10 min. Elution was monitored by measuring absorbance at 214 nm, and fractions were collected per 1 min. The eluted peptides were pooled into 20 fractions and vacuum-dried. Each fraction was resuspended in buffer A (2% ACN, 0.1% FA) and centrifuged at 20,000× *g* for 10 min. The supernatant was loaded onto a Thermo Scientific™ UltiMate™ 3000 UHPLC system (Thermo Scientific, Sunnyvale, CA, USA) equipped with a trap and an analytical column. The samples were loaded on a trap column at 5 μL/min for 8 min, and then eluted into the homemade nanocapillary C18 column (ID 75 μm × 25 cm, 3 μm particles) at a flow rate 300 nl/min. The gradient of buffer B (98% ACN, 0.1% FA) was increased from 5% to 25% in 40 min, and then increased to 35% in 5 min, followed by 2 min linear gradient to 80%, then maintenance at 80% B for 2 min, and, finally, returned to 5% in 1 min and equilibrated for 6 min. The peptides separated from nanoHPLC were subjected into the tandem mass spectrometry Q EXACTIVE HF X (Thermo Fisher Scientific, San Jose, CA, USA) for DDA (data-dependent acquisition) detection by nano-electrospray ionization. The parameters for MS analysis were listed as follows: electrospray voltage: 2.0 kV; precursor scan range: 350–1500 *m*/*z* at a resolution of 60,000 in Orbitrap; MS/MS fragment scan range: >100 *m*/*z* at a resolution of 15,000 in HCD mode; normalized collision energy setting: 30%; dynamic Exclusion time: 30 s; automatic gain control (AGC) for full MS target and MS2 target: 3 × 10^6^ and 1 × 10^5^, respectively. The MS/MS scan numbers following one MS scan: 20 most abundant precursor ions above a threshold ion count of 10,000.

### 2.5. Protein Quantification and Data Analysis

The raw MS/MS data were converted into MGF format, and the MGF files were searched by the local Mascot server against the database (Table S3). Besides, quality control was performed to determine if a reanalysis step was needed. An automated software, called IQuant, was applied to the quantification of proteins. All proteins with a false discovery rate (FDR) less than 1% proceeded to downstream analysis. Proteomic data are available by contacting the corresponding author.

### 2.6. Statistics

One-way ANOVA in SPSS 19.0 (SPSS Inc., Chicago, IL, USA) was used to compare the differences in rumen fermentation parameters and rumen epithelial morphology. The expression level of proteins involved in rumen ketogenesis was compared using Wilcoxon rank-sum test.

The criteria for the identification of differentially expressed proteins (DEPs) were according to false discovery rate (FDR) < 0.05 (Benjamini-Hochberg adjusted) and fold change (FC) > 1.8 (upregulated) or <0.56 (downregulated). The analyses of GO enrichment and KEGG pathways were carried out by KOBAS (version 3.0).

Bar charts were generated by GraphPad Prism 9 (https://www.graphpad-prism.cn/; accessed on 9 December 2022). Other figures, such as volcanic plots and heatmap, were generated by the R package ggplot2 (https://github.com/tidyverse/ggplot2; accessed on 9 December 2022).

## 3. Results

### 3.1. Growth Performance and Rumen Development 

**Figure 1:**
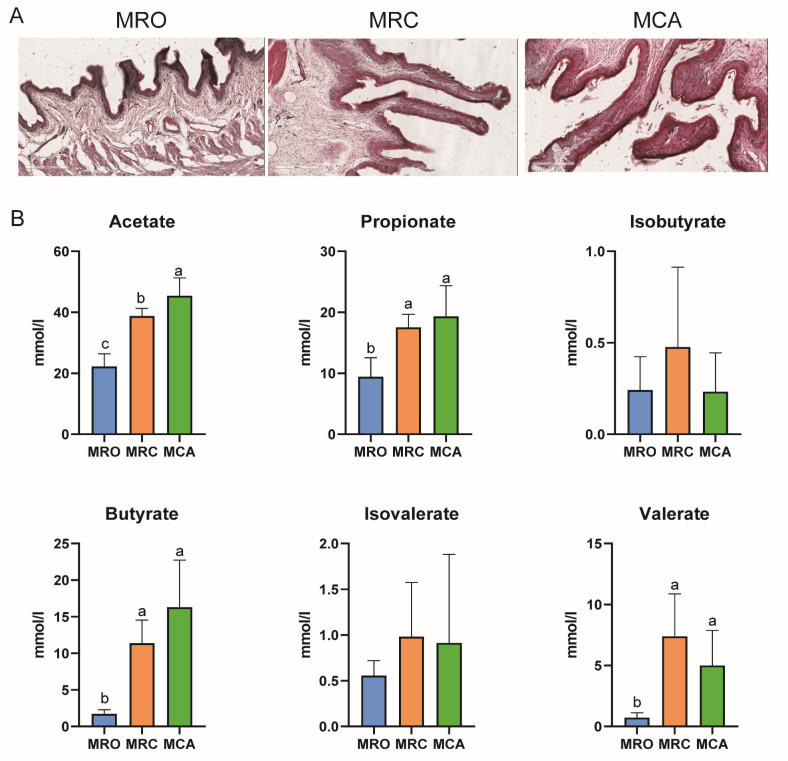
Rumen phenotypes in response to solid feed. (**A**) The development regularity of rumen epithelial morphology of goat kids that received MRO, MRC, and MCA diet. (**B**) The concentrations of acetate, propionate, isobutyrate, butyrate, and isovalerate of goat kids that received MRO, MRC, and MCA diet. Lowercase letters represent the level of significance.

In this study, we observed that solid feed supplementation could improve the growth performance of goat kids. Specifically, solid feed significantly increased the average daily gain (ADG), dry matter intake (DMI), and feed conversion rate (FCR) of goat kids in the MRC and MCA group (*p* < 0.05) (Table S4). In addition, compared with the MRO group, solid feed also significantly promoted the development of rumen epithelium in the MRC and MCA group, including an increased epithelium thickness and an increased height and width of the rumen papillae (*p* < 0.05) (Figure 1A, Table S5). The concentration of rumen VFA (mainly acetate, butyrate, and propionate) showed similar conditions (*p* < 0.05) (Figure 1B) [1,10].

### 3.2. The Identification of Differently Expressed Proteins (DEPs)

Based on analysis of iTRAQ data, a total of 114,401 (94,287 unique) spectra were identified. Of the 26,793 peptides, 24,609 peptides were unique. A total of 6003 proteins were identified with 1% FDR during the process of analysis (Table S6), and more than half of the proteins contained at least two peptides. The sequence coverage of most of the identified proteins was less than 20%, and approximately 60% of the proteins in the protein mass distribution were between 10 and 70 kDa. 

**Figure 2:**
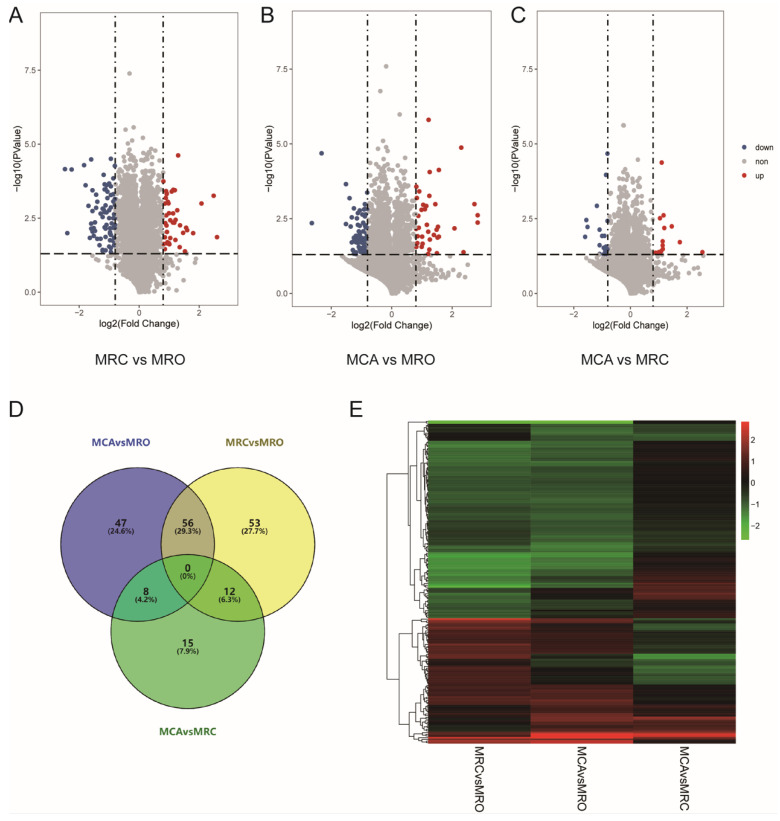
Characterization of Rumen epithelial protein. (**A**–**C**) The volcano plot of differently expressed proteins (DEPs) in pair-wise comparisons of (**A**) MRC vs. MRO, (**B**) MCA vs. MRO, and (**C**) MRC vs. MCA. (**D**) Wayne diagram of DEPs shared by the groups of MRC vs. MRO, MCA vs. MRO, and MRC vs. MCA. (**E**) The heatmap of the fold changes of proteins in the groups of MRC vs. MRO, MCA vs. MRO, and MRC vs. MCA.

For this experimental design with more than one replicate, proteins with 1.8-fold change (mean value of all comparison groups) or 0.56-fold change and a *p*-value (*t*-test of all comparison groups) less than 0.05 were defined as DEPs. In this experiment, in the MRC vs. MRO group, 121 kinds of DEPs were identified, of which 42 were upregulated and 79 were downregulated (Figure 2A). Further, 111 kinds of DEPs were detected in the MCA vs. MRO group, including 38 upregulated proteins and 73 downregulated proteins (Figure 2B). In addition, In the MCA vs. MRC group, a total of 35 DEPs were observed, including 15 upregulated proteins and 20 downregulated proteins. (Figure 2C). As shown in Figure 2D, 56 DEPs were shared with the MCA vs. MRO group and MRC vs. MRO group. The Heatmap also showed that, compared with the MCA vs. MRC group, there were more similar protein expression characteristics between the MCA vs. MRO group and MRC vs. MRO group (Figure 2E). 

To be specific, the DEPs of Shisa family member 4 (SHISA4) and Glutathione S-transferase (GSTA3) were all upregulated in both the MRC vs. MRO and MCA vs. MRO groups. In the MRC vs. MRO group, Semaphorin 3A (SEMA3A) and Tyrosine-protein kinase (FGR) were upregulated and emaphorin 3D (SEMA3D) and the NLR family pyrin domain containing 12 (NLRP12) were downregulated. In addition, Thyroid hormone receptor beta (THRB) and Semaphorin 3D (SEMA3D) were the most up- or down-regulated DEPs in the MCA vs. MRO group (Table 1 and Table 2).

Moreover, in the MCA vs. MRC group, the most upregulated and downregulated DEPs mainly included Olfactory receptor (LOC102188949, up), Nanos C2HC-type zinc finger 3 (NANOS3, up), Zinc finger protein 48 (ZNF48, down), and Interferon-induced protein with tetratricopeptide repeats 2 (IFIT2, down) (Table 3).

### 3.3. The Enrichment Analysis of DEPs According to Gene Ontology (GO)

To further explore the specific functional characteristics of the DEPs, we performed a GO enrichment analysis of the DEPs using David Bioinformatics Resources [18]. In general, in the MRC vs. MRO, the DEPs were significantly enriched in the 77 terms (*p* < 0.05), in which 23 terms were annotated with molecular function (MF), 15 terms were annotated with cellular component (CC), and 39 terms were annotated with biological process (BP) (Table S7). In the MCA vs. MRO, a total of 73 enriched terms were detected (*p* < 0.05), including 23 terms in MF, 18 terms in CC, and 32 terms in BP (Table S8). In the MCA vs. MRC group, 117 terms were enriched significantly (*p* < 0.05), consisting of 17 terms in MF, 21 terms in CC, and 79 terms in BP (Table S9).

**Figure 3:**
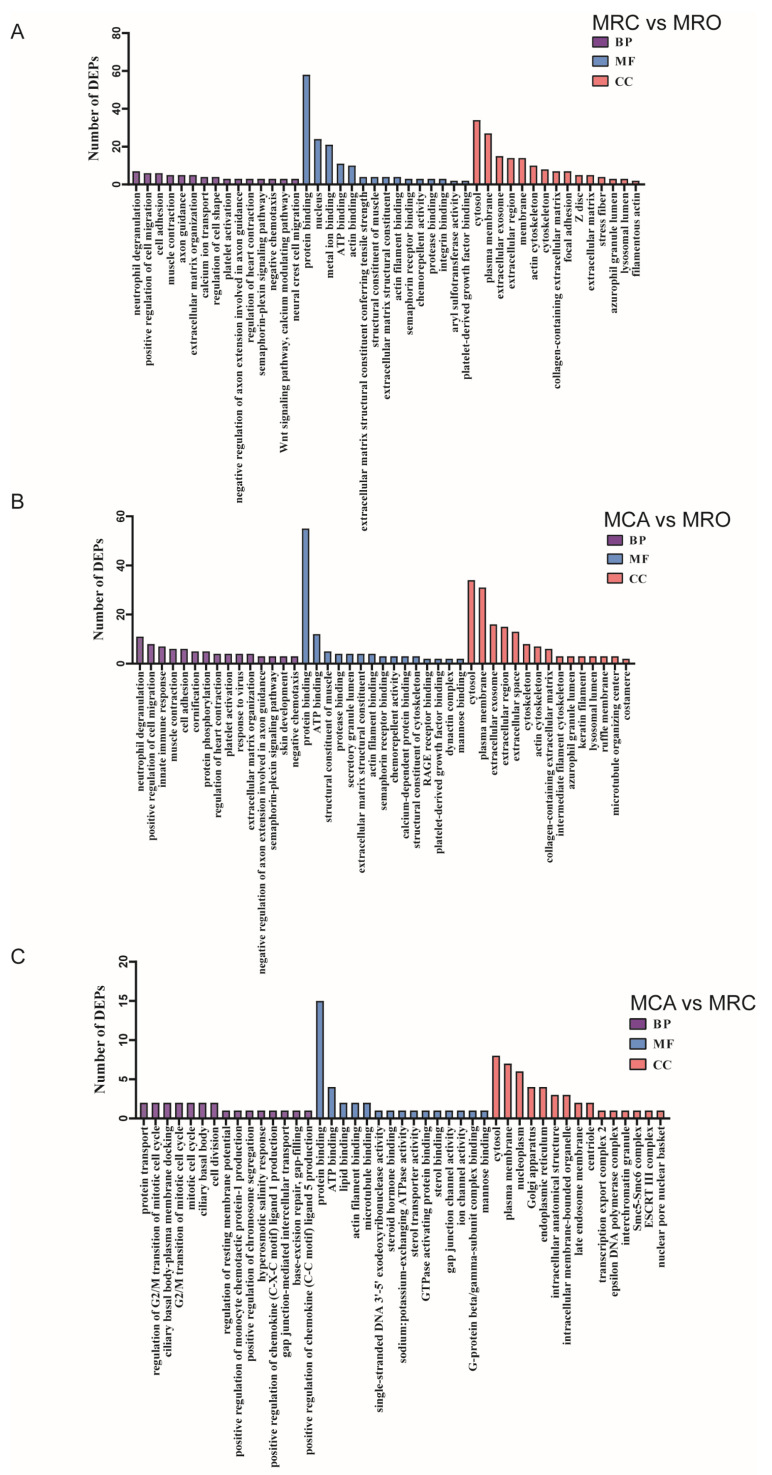
The bar charts of the top 15 significant GO terms in biological process (BP), cellular component (CC), and molecular function (MF) categories in the groups of (**A**) MRC vs. MRO, (**B**) MCA vs. MRO, and (**C**) MRC vs. MCA.

Moreover, the top 15 significant terms of each category (BP, CC, and MF) in the three comparison groups were screened out, respectively (Figure 3). In the category of BP, the DEPs in the two comparison groups (MRC vs. MRO and MCA vs. MRO) were enriched in similar terms, which was related to neutrophil degranulation (GO:0043312), positive regulation of cell migration (GO:0030335), muscle contraction (GO:0006936), and cell adhesion (GO:0007155) containing the DEPs of SEMA3A (upregulated), Recombinant Acetyl Coenzyme A Acetyltransferase 2 (ACAT2) (upregulated), Caldesmon 1 (CALD1) (downregulated), and Protein kinase X-linked (PRKX) (upregulated) (Figure 3A,B). In the category of MF, protein binding (GO:0005515) was the most enriched term, and was shared by all three comparison groups of the DEPs of acetyl-Coenzyme A acyltransferase 2 (ACAA2) (upregulated), Actin-related protein 10 (ACTR10) (upregulated), and Phospholipid-transporting ATPase (ATP11B) (upregulated). Moreover, the term of ATP binding (GO:0005524) showed a similar condition, and was enriched by the DEPs of Sodium/potassium-transporting ATPase subunit alpha (ATP1A3) (upregulated), Phospholipid-transporting ATPase (ATP11B) (upregulated), Protein kinase X-linked (PRKX) (upregulated), and RNA 3′-terminal phosphate cyclase (RTCA) (upregulated) (Figure 3). In the term of CC, the DEPs in the two comparison groups (MRC vs. MRO and MCA vs. MRO) were both significantly enriched in the terms of cytosol (GO:0005829), plasma membrane (GO:0005886), extracellular exosome (GO:0070062), and extracellular region (GO:0005576), including the DEPs of 3-hydroxy-3-methylglutarate-CoA lyase (HMGCL) (upregulated), Sulfotransferase (SULT1B1) (upregulated), Chloride anion exchanger (SLC26A3) (upregulated), Protein kinase C (PRKCB) (downregulated), and Monocarboxylate transporter 1 (SLC16A1) (upregulated) (Figure 3A,B).

### 3.4. The KEGG Enrichment Analysis of DEPs

**Figure 4:**
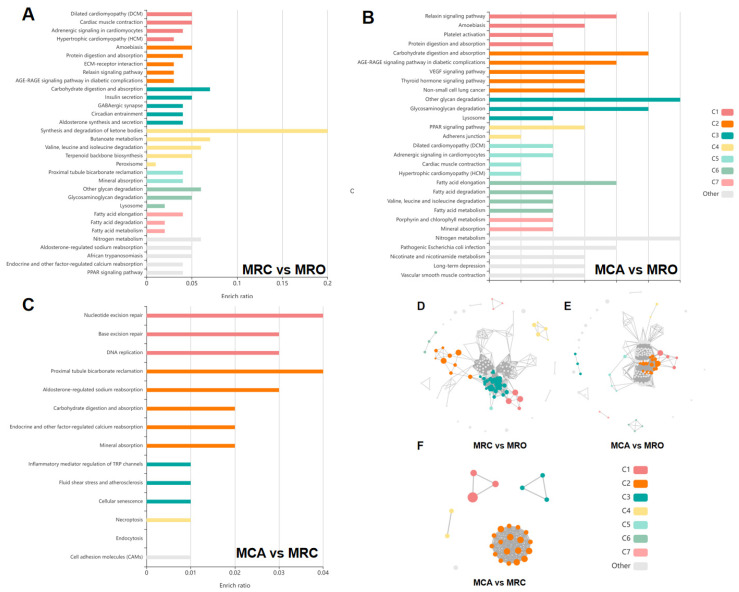
Kyoto encyclopedia of genes and genomes (KEGG) analysis of DEPs in the groups of (**A**,**D**) MRC vs. MRO, (**B**,**E**) MCA vs. MRO, and (**C**,**F**) MRC vs. MCA. (**A**–**C**). Each row represents an enriched function, and the length of the bar represents the enrichment ratio, which is calculated as “input gene number”/“background gene number.” The color of the bar is the same as the color in the circular network above, which represents different clusters. For each cluster, if there are more than five terms, the top five with the highest enrichment ratio will be displayed. (**D**–**F**) Each bubble represents an enriched function, and the size of the bubble from small to large: The color of the bar is the same as the color in the circular network, which represents different clusters. For each cluster, if there are more than five terms, the top five with the highest enrichment ratio will be displayed.

To understand the effects of the DEPs on the related pathways after the introduction of solid feed, we performed a KEGG pathway enrichment analysis of the DEPs. In the MRC vs. MRO group, the DEPs were enriched in 32 pathways, most of which belonged to the categories of organismal systems and metabolism. The network cluster analysis showed that the pathways with a high enrichment ratio, including Carbohydrate digestion and absorption (chx04973), Synthesis and degradation of ketone bodies (chx00072), and Butanoate metabolism (chx00650), were the critical nodes connecting the respective clusters (Figure 4A,D). In the MCA vs. MRO group, the DEPs were enriched in 29 pathways. Similarly, organismal systems and metabolism were the main categories. The pathways of carbohydrate digestion and absorption (chx04973), other glycan degradation (chx00511), Glycosaminoglycan degradation (chx00531), Fatty acid elongation (chx00062), Fatty acid degradation (chx00071), and PPAR signaling (chx03320) were determined to be important nodes, as shown by their high enrichment ratio (Figure 4B,E). In addition, the pathways enriched by DEPs in the MCA vs. MRC group were mainly enriched in the pathways of Nucleotide excision repair (chx03420) and Proximal tubule bicarbonate reclamation (chx04964) (Figure 4C,F).

### 3.5. The PPI Analysis of DEPs

**Figure 5:**
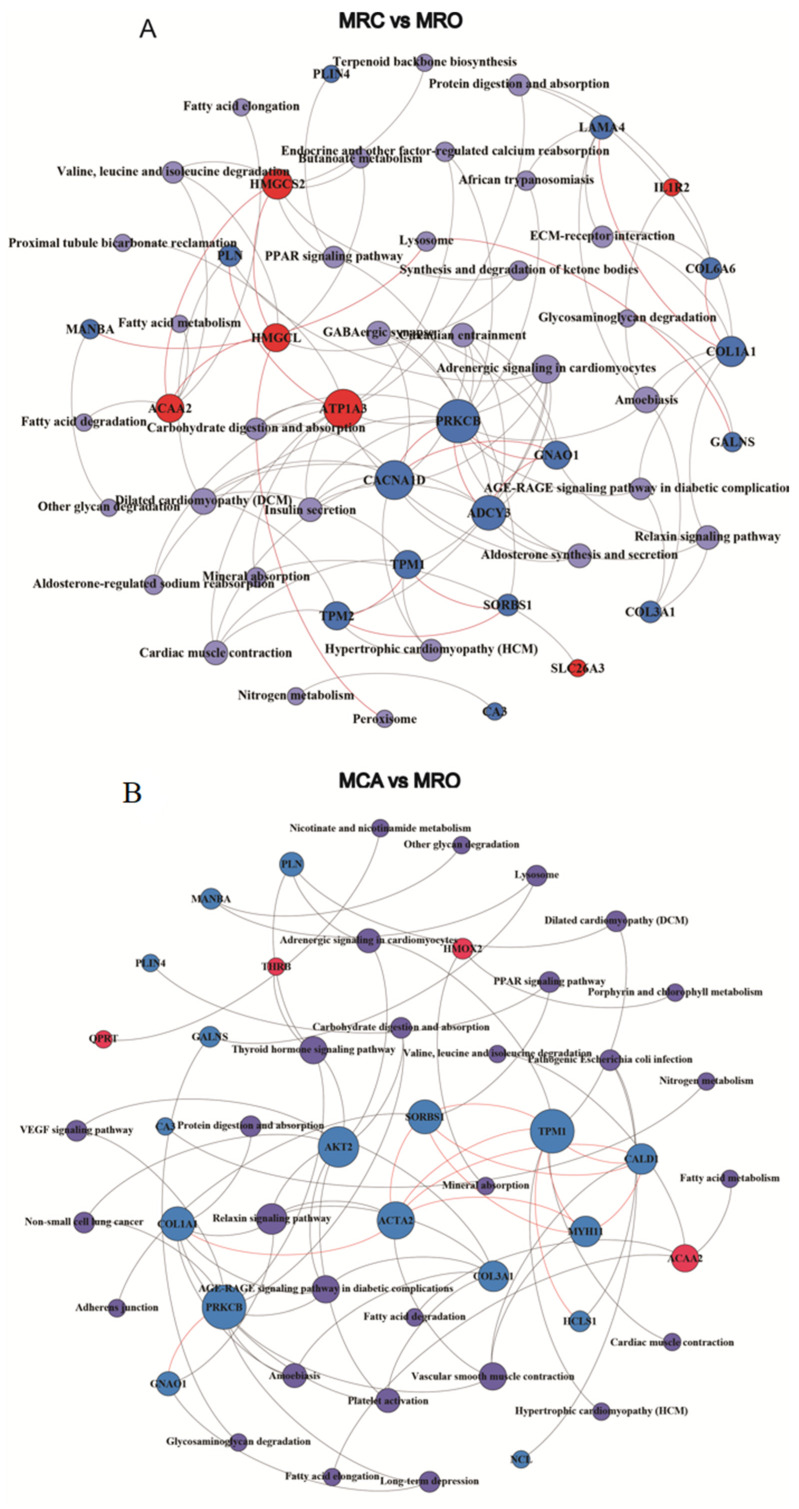
PPI network analysis according to STRING database and KEGG pathway enriched in DEPs in the groups of (**A**) MRC vs. MRO and (**B**) MCA vs. MRO.

In order to further understand the interaction between DEPs and their related functional pathways, PPI network analysis was performed according to the STRING database and KEGG results (Figure 5). In the MRC vs. MRO group, a total of 23 DEPs were identified as key nodes which showed various connections in the network, including 6 upregulated DEPs (SLC26A3, Interleukin 1 receptor type 2 (IL1R2), HMGCS2, HMGCL, ATP1A3, and ACAA2) and 16 downregulated DEPs (Tropomyosin 1 and 2 (TPM1 and 2), Sorbin and SH3 domain containing 1 (SORBS1), PRKCB, Cardiac phospholamban (PLN), Perilipin 4 (PLIN4), Beta-mannosidase (MANBA), Laminin subunit alpha 4 (LAMA4, G protein subunit alpha o1 (GNAO1), Chondroitinsulfatase (GALNS), Collagen type VI alpha 6 chain (COL6A6), Collagen type III alpha 1 chain (COL3A1), Collagen type I alpha 1 chain (COL1A1), Voltage-dependent L-type calcium channel subunit alpha (CACNA1D), Carbonic anhydrase (CA3), and Adenylate cyclase type 3 (ADCY3)) (Figure 5A). In the MCA vs. MRO group, 19 DEPs were identified as key nodes, including four upregulated DEPs (ACAA2, THRB, Heme oxygenase (biliverdin-producing) (HMOX2), and Nicotinate-nucleotide pyrophosphorylase (QPRT)) and 15 downregulated DEPs (Hematopoietic cell-specific Lyn substrate 1 (HCLS1), Non-specific serine/threonine protein kinase (AKT2), GNAO1, PLIN4, CA3, Nucleolin (NCL), COL1A1, MANBA, MYH11, PRKCB, CALD1, GALNS, TPM1, SORBS1, and PLN) (Figure 5B). It is worth noting that many DEPs in the networks, such as ACAA2, HMGCS2, ATP1A3, HMGCL, etc., were associated with metabolism pathways.

### 3.6. Rumen Ketogenesis in Response to Solid Feed Supplementation

**Figure 6:**
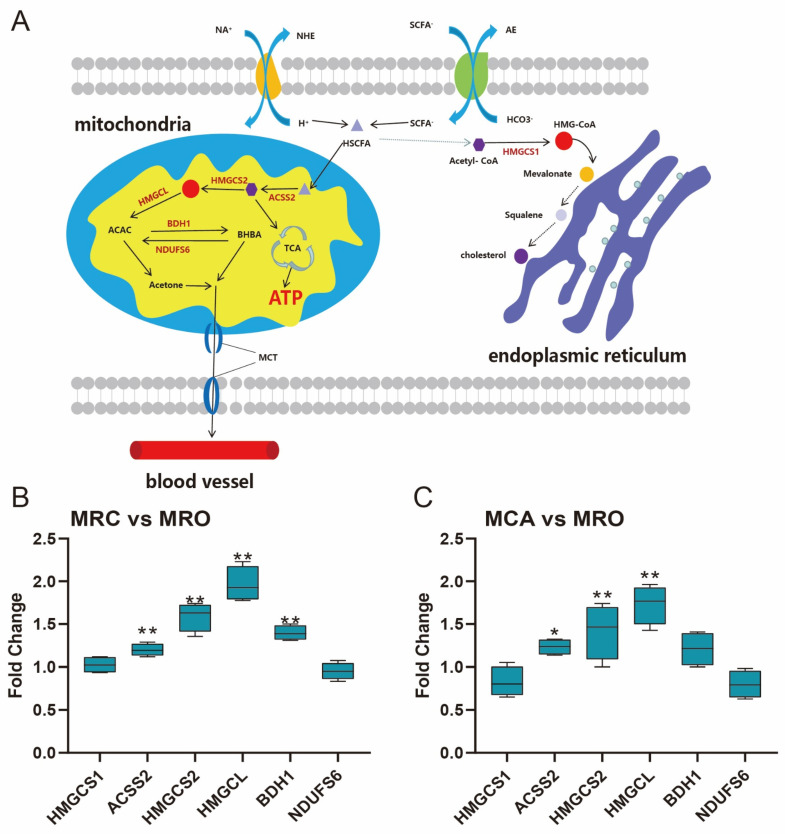
The metabolic pathway for ketone body by conversion from SCFA. (**A**) the metabolic profile of rumen ketogenesis, this figure was drawn with reference to a published paper [19]; (**B**,**C**) The protein expression of key enzymes in the process of rumen ketogenesis in the groups of (**B**) MRC vs. MRO and (**C**) MCA vs. MRC; * 0.01 < *p* < 0.05; ** 0.001 < *p* < 0.01.

In this study, considering the higher concentration of VFAs and the length of rumen papillae in response to solid feed supplementation, we identified the protein expression of key enzymes in the process of rumen ketogenesis to characterize the efficiency of energy production in the development of rumen (Figure 6A). Compared with the MRO group, the protein expressions of the enzymes including Acetyl CoA synthetase (ACSS2), HMGCS2, HMGCL, and D-beta-hydroxybutyrate Dehydrogenase (BDH1) were significantly higher (*p* < 0.05) in the MRC group (Figure 6B). Similarly, in the MCA group, the protein expressions of ACSS2, HMGCS2, and HMGCL were also higher than in the MRO group (*p* < 0.05) (Figure 6C).

## 4. Discussion

### 4.1. Rumen Fermentation Parameters and Papillae Development

In the study, the diet with solid feed supplantation increased the concentrations of VFAs in the rumen and promoted the growth of rumen papillae [1,4]. On the one hand, the introduction of solid feed provides rich fermentation substrates for nutrient-decomposing bacteria in the rumen [20,21]. The VFAs produced by rumen microbiota became the primary energy supply for the development of rumen epithelium [22,23]. Moreover, the physical friction of the solid feed against the rumen wall further stimulates the growth of the rumen papillae [7,9]. A larger rumen epithelial surface had higher metabolic efficiency for nutrients, including VFAs and microbial protein, to meet the more exuberant growth demands of the host [24]. In addition, the metabolic pattern of rumen epithelium is also regulated by solid feed. Thus, we further discussed the effect of solid feed supplementation on rumen epithelial proteins from the view of biochemistry and metabolism in detail.

### 4.2. Cell Development of Rumen Epithelium

In this study, some GO terms were associated with the cell development of rumen epithelium, including extracellular matrix structural constituent conferring tensile strength, extracellular matrix organization, extracellular matrix structural constituent, collagen-containing extracellular matrix, plasma membrane, extracellular exosome, extracellular region, etc., which contained the DEPs of COL3A1, Collagen type XVI alpha 1 chain (COL16A1), COL6A6, COL1A1, LAMA4, FGR, formin-like 3 (FMNL3), etc.

The complete construction of rumen epithelial cells was a prerequisite for the rumen metabolism [25]. Previous studies have proven that COL3A1, COL16A1, COL6A6, and COL1A1 were involved in the regulation of blood vessel development and construction in the gut epithelium. LAMA4, encoding a secreted glycoprotein, was found to influence cell adhesion, which plays a key role in cell proliferation, the maintenance of activity, differentiation, and migration [26] In this study, these DEPs were decreased in response to solid feed. FMNL3 was identified as an effector of Rho GTPases, contributing to different cellular actin cytoskeleton structures by its ability to polymerize straight actin filaments at the barbed end [27]. FGR was a critical covalent modification and occurs in multicellular organisms as a result of intercellular communication during the maintenance of adult tissues [28]. In contrast, FMNL3 and FGR were increased with solid feed supplantation in this study. According to our findings, we thought solid feed introduction promoted the development of rumen epithelium by regulating the expression of proteins related to cell growth and integrity. As we know, solid feed is rich in nutrients including starch, fat, protein, etc. The complex and diverse microbial communities in the rumen can efficiently ferment them into VFAs and high-quality microbial proteins, which are energy substrates that can be directly absorbed and utilized by rumen epithelial cells [4]. Hence, we hypothesized that the supplement of solid feed provided these material bases for the rumen growth of goat kids, and promoted the faster development of rumen epithelial cells, including cell adhesion and frame construction. However, the internal mechanism of these regulations still needs to be confirmed in further study.

### 4.3. Metabolism of Rumen Epithelium

As we know, with an increase in age, the main digestive organs of young ruminants are transferred from the hindgut to the mature rumen [29,30]. VFAs also replace glucose as the main energy substance for their growth [21,31]. In this study, the metabolism pathways associated with VFAs, including fatty acid elongation, fatty acid degradation, butanoate metabolism and synthesis, and the degradation of ketone bodies, were the most significant, which could be attributed to the increasing concentration of butyrate after solid feed supplementation. The rumen epithelial mitochondria is the main site of butyric acid metabolism. The energy produced during the conversion of butyrate to ketone bodies is the main source of energy for epithelial growth [32]. Hence, the ability of the rumen to produce ketone bodies (mainly β-hydroxybutyrate acid (BHBA)) is considered to be an important marker of rumen development [33]. In terms of biochemical steps, the expression of enzyme proteins involved in VFA metabolism and ketone body synthesis showed a significant increase, including ACSS2, HMGCL, HMGCS2, ACAA2, and BDH1. Acetyl-CoA is an important pivotal substance in energy metabolism which is not only a carbon source for the synthesis of ketones or cholesterol, but also participates in the tricarboxylic acid cycle (TCA) for energy production [34]. As the synthetase of acetyl-CoA, the increased expression of ACSS2 protein meant the active performance of energy metabolism in the rumen epithelium. The significant GO term of ATP binding also corresponded to this result. HMGCS2 is the rate-limiting enzyme involved in the generation of ketone bodies from lipids in the rumen [35]. Recent studies have also reported that the accumulation of BHBA and the increase in HMGCS2 expression in ketogenesis strengthened the ability of gut epithelial cells to proliferate, differentiate, and maintain gut homeostasis [36,37]. BDH1 is the last enzyme of rumen ketogenesis, catalyzing the reversible reduction of acetoacetate (ACAC) to BHBA [38]. ACAA2 is an enzyme of the thiolase family that performs the function of mitochondrial fatty acid extension and degradation by catalyzing the last step of the β-oxidation pathway. ACAA2 often showed a high expression in tissues with active lipid metabolism such as mammary gland and liver [39]. In addition, some DEPs associated with the transport of fatty acids were also identified. The protein expressions of two transporters (SLC26A3 and SLC16A1) were increased with the introduction of solid feed. SLC26A3, as an anion exchanger, mediates apical Cl^−^/HCO_3_ exchange in intestinal epithelia [40]. SLC26A3 was also observed in the rumen epithelium, where its main function is to transport short chain fatty acid (SCFA) [41]. The surface of rumen epithelium maintained an acidic environment due to the accumulation of a large number of VFAs, and SLC26A3 promotes the absorption of short-chain fatty acids (SCFA-) through SCFA-/HCO3- exchange [42]. SLC16A1 (MCT1) has been suggested to be a rumen transporter, mediating SCFA, ketone bodies, and lactic acid [43]. After SCFA have reached the mitochondrial-rich layer cells by the diffusion of functional syntheses, they undergo oxidative metabolism, and subsequent metabolites are transported to the blood by SLC16A1. Therefore, rumen epithelium utilized the high concentration of VFAs fermented from solid feed via increasing the expression of proteins related to VFA metabolism and transport. One study also showed that the direct infusion of sodium butyrate to neonatal lambs could promote the expression of genes related to VFA absorption and ketone body metabolism in rumen epithelium, which is consistent with our results [23]. These results suggested that butyrate fermented from solid feed is the most direct factor which promotes the development of rumen epithelium.

In contrast, we observed that the significant pathways of carbohydrate digestion and absorption were inactive, which was associated with two downregulated DEPs (AKT2 and PRKCB). Protein kinase C (PKC), encoded by the PRKCB1, is a family of serine- and threonine-specific protein kinases which could be involved in endothelial cell apoptosis, intestinal sugar absorption, etc. AKT2 is associated with the positive regulation of glucose import, and the knockout or decreased expression of Akt2 could inhibit the uptake and utilization of glucose by cells [44], enhance the resistance to fat accumulation, and improve the metabolic efficiency of lipids [45]. The energy metabolism process of ruminants changed from glucose-based into volatile acid-based with the increase in age and the development of rumen [46]. Combined with our results, the introduction of solid feed accelerated this process, and the high concentration of VFAs in the rumen dominated the metabolic pattern of rumen epithelium, which inhibited the metabolism of glucose and promoted the utilization of VFAs in the mitochondria via decreasing the expression of AKT2 and PRKCB.

### 4.4. Signal Transduction of Rumen Epithelium

In this study, PPAR signaling pathways were significantly enriched with upregulated protein (HMGCS2) and downregulated protein (Perilipin 4 (PLIN4) in both the groups of MRC/MCA vs. MRO. The induction of HMGCS2 is mainly controlled by the positive regulation of peroxisome proliferator-activated receptors (PPARs) [47]. SCFAs, as efficient ligands, could effectively activate the PPAR signaling pathway and promote the expression of the target gene HMGCS2 [48]. PLIN4, as a lipid droplet protein (LDP), exists widely in oxidized tissue, and the inactivation of PLIN4 could promote the metabolism of lipids for preventing their accumulation [49]. Thus, we hypothesized that VFAs in the rumen as ligand could activate PPAR signaling pathways, which would regulate the expression of HMGCS2 and PLIN4 for promoting fatty acid metabolism.

## 5. Conclusions

In summary, solid feed as an initiating agent increased the growth performance of goat kids, enhanced the ability of rumen fermentation, and promoted the development of rumen epithelium. In addition, according to the previous studies on the transcriptional level, we further explored the effect of solid feed supplantation on the development of rumen epithelium at the protein level. As the results indicate, solid feed promoted the development of rumen epithelium by changing the expression of proteins related to cell construction, fatty acid metabolism, and PPAR signal transduction. The ketone body synthesis pathway might be one important pathway which is activated in response to solid feed supplementation, which could provide abundant energy for rumen development. Our findings broaden the theoretical knowledge of the intrinsic mechanisms of rumen development in young ruminants.

## Figures and Tables

**Table 1:** The DEPs in the rumen epithelium of goat kids in the MRC group compared with the MRO group.

Protein_ID	Protein Name	Gene Symbol	Mass	Protein_Coverage	Uniq_Pep	Uniq_Spec	log2Fold Change
W5PDY2	Uncharacterized protein	-	261,849.67	0	1	1	2.59
A0A452EGR5	Shisa family member 4	SHISA4	26,983.28	0.03	1	2	2.48
W5Q115	semaphorin 3A	SEMA3A	95,951.2	0.02	1	1	2.08
W5NZJ6	Tyrosine-protein kinase	FGR	52,840.21	0.03	1	1	1.8
W5P9E2	SERPIN domain-containing protein	LOC101103612	47,177.77	0.18	1	1	1.6
A0A452FDU1	Phosphodiesterase	PDE6B	97,921.12	0.01	1	1	1.57
W5PMM4	Aldo_ket_red domain-containing protein	LOC106990122	37,134.33	0.36	1	1	1.53
A0A452F710	RNA 3′-terminal phosphate cyclase	RTCA	39,748.46	0.04	1	1	1.46
W5P6R6	Ubiquitin carboxyl-terminal hydrolase	USP27X	59,865.01	0.04	1	1	1.36
W5P8B8	Zinc finger protein 48	ZNF48	51,013.33	0.02	1	1	1.35
A0A452FGG7	Sulfotransferase	SULT1C2	34,285.13	0.08	1	1	1.3
A0A452DK46	Carbonic anhydrase	LOC102184245	29,121.58	0.41	1	12	1.27
Q0PD85	Monocarboxylate transporter 1	SLC16A1	35,188.38	0.05	1	2	1.21
W5QJ02	Dehydrogenase/reductase 7	DHRS7	37,262.55	0.31	1	2	1.21
A0A452DKF9	4HBT domain-containing protein	-	13,848.09	0.26	1	1	1.19
W5PJY0	ER membrane protein complex subunit 1	EMC1	112,940.55	0.12	1	1	1.17
A0A452ELD7	BPI fold containing family A member 2	BPIFA2	28,888.13	0.12	2	2	1.14
W5QEK2	Tubulin tyrosine ligase like 4	TTLL4	133,873.12	0	1	1	1.13
W5P9M9	SERPIN domain-containing protein	LOC101103862	44,569.58	0.23	1	1	1.12
A0A452EBL1	Dihydrodiol dehydrogenase 3	LOC102177638	37,351.57	0.33	1	1	1.08
W5QAX2	HDAC_interact domain-containing protein	SIN3B	117,126.51	0.01	1	1	1.04
W5P1M4	Acetyl-CoA acyltransferase 2	ACAA2	42,086.45	0.41	1	3	1.04
A0A452G3K1	Actin related protein 10	ACTR10	44,622.44	0.02	1	1	1.01
A0A452F7V6	Centrin 2	CETN2	19,100.59	0.08	1	1	0.99
A0A452E3H3	UDP-glucuronosyltransferase	LOC102172432	61,207.33	0.22	2	2	0.99
A0A452GAC9	3-hydroxy-3-methylglutarate-CoA lyase	HMGCL	34,621.11	0.3	8	24	0.98
W5Q6A5	Charged multivesicular body protein 2B	CHMP2B	23,990.35	0.03	1	1	0.96
A0A452FP65	UDP-glucuronosyltransferase	LOC108633190	61,515.7	0.08	1	6	0.94
W5QHL0	Phospholipid-transporting ATPase	ATP11B	125,527.01	0.01	1	1	0.93
A0A452FF33	Sodium/potassium-transporting ATPase subunit alpha	ATP1A3	108,265.03	0.16	1	1	0.93
W5PUH3	Interleukin 1 receptor type 2	IL1R2	45,313.01	0.04	1	1	0.92
W5PAD4	Chloride anion exchanger	SLC26A3	84,603.08	0.05	4	7	0.92
A0A452EX18	Serine and arginine rich splicing factor 5	SRSF5	31,584.45	0.03	1	1	0.91
W5P699	M-phase phosphoprotein 9	MPHOSPH9	133,521.74	0.02	1	1	0.9
W5PLM4	Aldo_ket_red domain-containing protein	-	32,650.38	0.33	1	1	0.9
D4P8J3	3-hydroxy-3-methylglutaryl coenzyme A synthase	HMGCS2	57,306.89	0.31	13	105	0.89
A0A452EWC8	Monocarboxylate transporter 1	SLC16A1	54,633.26	0.07	2	3	0.87
A0A452FCI0	Zinc finger protein 416	LOC102179804	67,775.27	0.06	1	1	0.86
A0A452DQX5	Sulfotransferase	SULT1B1	34,876.74	0.11	3	3	0.86
W5QAL6	Formin like 3	FMNL3	117,941.04	0.01	1	1	0.86
W5PGI1	Protein kinase X-linked	PRKX	35,458.36	0.06	1	1	0.82
A0A452DRA0	Trichohyalin	TCHH	188,388.47	0.05	2	3	0.81
W5PZZ4	Homeobox domain-containing protein	-	11,162.59	0.12	1	1	−0.81
W5QHV0	SET and MYND domain containing 1	SMYD1	57,333.11	0.03	1	2	−0.81
W5PFB6	CD177 molecule	CD177	47,924.93	0.03	1	1	−0.81
A0A452F2B2	G protein subunit alpha o1	GNAO1	40,653.28	0.07	1	1	−0.81
A0A452DVI8	Anti-Muellerian hormone type-2 receptor	AMHR2	61,515.15	0.01	1	2	−0.81
A0A452FAG1	Laminin subunit alpha 4	LAMA4	205,009.31	0.15	21	32	−0.81
K4PF82	RNA-binding protein with serine-rich domain 1	RNPS1	34,157.69	0.13	2	2	−0.81
A0A452FD11	Tropomyosin 1	TPM1	32,731.66	0.23	3	10	−0.84
A0A452FTD0	Ig-like domain-containing protein	-	19,018.29	0.09	2	2	−0.84
A0A452E1D7	Ankyrin repeat and SOCS box containing 7	ASB7	36,426.64	0.02	1	1	−0.84
W5PX13	Gap junction alpha-3 protein	GJA3	35,165.12	0.03	1	2	−0.84
A0A452FV55	Collagen type XVI alpha 1 chain	COL16A1	147,706.13	0.01	1	1	−0.84
A0A452FBG8	Smoothelin	SMTN	98,772.31	0.09	6	7	−0.86
A0A452EMX2	Tropomyosin 2	TPM2	32,994.61	0.24	2	3	−0.86
A0A452G0P9	Parvin alpha	PARVA	46,577.9	0.11	3	5	−0.86
A0A452EAY9	A0A452EAY9	-	28,201.62	0.29	1	1	−0.86
W5NYX4	NACHT domain-containing protein	-	104,305.02	0.01	1	1	−0.86
Q4LBD9	Q4LBD9	ovar-MHCI-H10	41,373.53	0.12	2	2	−0.86
A0A452EYA6	Protein kinase C	PRKCB	78,062.58	0.03	1	1	−0.92
W5PIF9	Transforming growth factor beta 1 induced transcript 1	TGFB1I1	51,299.58	0.04	2	3	−0.94
A0A452EUR3	Guanylate cyclase	-	46,878.37	0.02	1	1	−0.94
A0A452E7G7	Family with sequence similarity 71 member A	FAM71A	63,850.57	0.02	1	1	−0.94
W5Q4S0	Collagen type III alpha 1 chain	COL3A1	139,701.25	0.08	8	35	−0.94
A0A452G5Y9	Netrin 3	NTN3	63,599.98	0.01	1	1	−0.94
W5PW78	Sema domain-containing protein	SEMA4C	81,534.11	0.02	1	1	−0.94
W5Q2A6	EF-hand domain family member B	EFHB	97,299.48	0.01	1	1	−0.94
A0A452G7W8	Family with sequence similarity 110 member C	FAM110C	30,168.37	0.03	1	1	−0.97
A1YZ35	Mimecan	OGN	34,450.92	0.29	8	26	−0.97
A0A452EWQ2	SPEM family member 2	SPEM2	56,745.55	0.02	1	1	−0.97
A0A452EV55	A0A452EV55	-	213,096.55	0	1	1	−0.97
A0A452FFP6	Beta-mannosidase	MANBA	102,118.69	0.03	1	1	−0.97
A0A452G7S1	HLA class II histocompatibility antigen, DM beta chain	LOC102187998	29,197.63	0.06	1	1	−1
A0A452F535	General transcription factor IIF subunit 2	-	28,635.95	0.06	1	1	−1
A0A452ECP7	Carbonic anhydrase	CA3	29,707.9	0.25	5	8	−1
W5NYU5	USP domain-containing protein	USP43	112,010.29	0.01	1	2	−1
A0A452F0J4	SPHK1 interactor, AKAP domain containing	SPHKAP	182,094.96	0.01	1	2	−1.06
A0A452G4N4	Adenylate cyclase type 3	ADCY3	130,124.24	0.02	1	1	−1.06
A0A452F637	Collagen type VI alpha 6 chain	COL6A6	246,602.86	0	1	1	−1.09
A0A452G885	A0A452G885	KRT4	56,270.57	0.39	13	30	−1.09
A0A452E7J1	Olfactory receptor	OR1G1	35,499.99	0.13	1	1	−1.09
A0A452FWB4	Corepressor interacting with RBPJ, 1	CIR1	47,287.89	0.02	1	2	−1.12
A0A452FHU9	Collagen type I alpha 1 chain	TPM1A1	139,952	0.18	15	179	−1.12
W5QHQ7	Nucleolin	NCL	73,334.54	0.15	1	1	−1.12
A0A452FMJ8	Synaptopodin 2	SYNPO2	115,845.7	0.24	1	1	−1.12
W5PY97	HP domain-containing protein	SVIL	246,128.41	0.02	1	1	−1.12
A0A452F3X3	Sorbin and SH3 domain containing 2	SORBS2	138,370.92	0.17	12	20	−1.15
Q6S5L3	Major histocompatibility class II DQA1	Cahi-DQA1	28,131.18	0.09	2	2	−1.15
A0A0H5FSL3	MHC class I antigen	Ovar-I	17,440.29	0.17	1	1	−1.15
A0A452G1W6	Solute carrier family 9 member C2 (putative)	SLC9C2	129,059.02	0	1	2	−1.15
A0A452FBH2	2′-5′ oligoadenylate synthase	OAS1	41,810.87	0.13	1	2	−1.18
A0A452E3D3	Perilipin 4	PLIN4	126,234.86	0.13	12	16	−1.18
A0A224ATJ6	Olfactory receptor	OR4X2	29,701.85	0.1	1	1	−1.22
A0A452EL11	Chondroitinsulfatase	GALNS	55,667.92	0.02	1	1	−1.22
A0A1S6YF29	ATP synthase subunit a	ATP6	24,695.89	0.06	1	1	−1.22
W5PA36	Zinc finger CCCH-type containing 7A	ZC3H7A	112,014.91	0.01	1	1	−1.22
A0A452E3Q2	Argonaute RISC catalytic component 2	AGO2	97,447.94	0.09	1	1	−1.29
A0A452FXD3	GMP reductase	GMPR	34,625.42	0.07	1	1	−1.29
A0A452EA30	PDZ and LIM domain 7	PDLIM7	52,633.91	0.24	1	1	−1.32
W5Q756	WH2 domain-containing protein	JMY	108,165.74	0.02	1	1	−1.4
W5PI04	Rab-GAP TBC domain-containing protein	TBC1D13	37,975.02	0.03	1	1	−1.4
W5PD82	Caldesmon 1	CALD1	89,883.93	0.14	9	20	−1.43
A0A452E4C4	ADAM metallopeptidase with thrombospondin type 1 motif 16	ADAMTS16	143,816.25	0	1	1	−1.43
A0A452FL85	Profilin	PFN2	15,346.37	0.23	2	5	−1.47
A0A452DQP4	Polycystin 1 like 2	PKD1L2	272,917.19	0	1	1	−1.47
A0A452FL64	Resistin	RETN	12,192.84	0.24	1	1	−1.51
W5Q6L1	IF rod domain-containing protein	KRT85	40,944.41	0.05	1	1	−1.51
W5NS84	Ret finger protein-like 4A	LOC101116932	32,111.24	0.03	1	1	−1.56
Q9XSQ8	MAP28 protein	map28	18,000.42	0.08	1	1	−1.56
W5NV41	RING-type domain-containing protein	RNF175	38,416.48	0.07	1	1	−1.56
A0A452E9K4	Cardiac phospholamban	PLN	6229.38	0.21	1	1	−1.6
R9WH56	MHC class I antigen	OLA-I	41,077.25	0.1	1	1	−1.6
A0A452EH97	Sorbin and SH3 domain containing 1	SORBS1	142,840.96	0.15	1	3	−1.6
A0A452EKJ0	Glycerophosphodiester phosphodiesterase domain containing 3	GDPD3	36,818.99	0.04	1	1	−1.64
W5QB39	Glutamate receptor interacting protein 2	GRIP2	110,290.75	0.01	1	1	−1.69
A0A452FA76	Voltage-dependent L-type calcium channel subunit alpha	CACNA1D	239,629.01	0.01	1	1	−1.79
A0A452F4F7	Sorbin and SH3 domain containing 2	SORBS2	17,636.7	0.26	1	1	−1.84
A0A452EWT8	NLR family pyrin domain containing 12	NLRP12	119,902.48	0.01	1	1	−2.25
A0A452F5B1	Semaphorin 3D	SEMA3D	90,605.53	0.04	1	1	−2.4
A0A068B4V9	Glutathione S-transferase	GSTA3	25,485.39	0.29	1	1	−2.47

**Table 2:** The DEPs in the rumen epithelium of goat kids in the MCA group compared with the MRO group.

Protein_ID	Protein Name	Gene Symbol	Mass	Protein_Coverage	Uniq_Pep	Uniq_Spec	log2Fold Change
Q28571	Thyroid hormone receptor beta	THRB	47,694.59	0.02	1	1	2.84
A0A452EGR5	Shisa family member 4	SHISA4	26,983.28	0.03	1	2	2.84
A0A452DTY3	Olfactory receptor	LOC102188949	35,991.46	0.11	1	1	2.74
A0A452EZP2	Anoctamin	ANO9	89,634.08	0.01	1	2	2.38
W5Q115	Semaphorin 3A	SEMA3A	95,951.2	0.02	1	1	2.3
A0A452EMA9	Oocyte-secreted protein 1	LOC102171358	15,888.39	0.07	1	1	2.08
A0A452FJ40	Membrane cofactor protein	LOC102169209	37,010.88	0.05	1	1	1.56
W5P5J3	Adhesion G protein-coupled receptor A3	ADGRA3	139,118.94	0	1	1	1.56
W5P9E2	Serpin B3-like	LOC101103612	47,177.77	0.18	1	1	1.53
W5QJ02	Dehydrogenase/reductase 7	DHRS7	37,262.55	0.31	1	2	1.52
A0A452FNE3	PDZ domain containing 3	PDZD3	52,094.46	0.04	1	2	1.5
A0A452DVC9	Nanos C2HC-type zinc finger 3	NANOS3	19,372.36	0.05	1	1	1.49
A0A452F7Y9	Fer-1 like family member 6	FER1L6	206,943	0.01	1	1	1.44
W5NZJ6	Tyrosine-protein kinase	FGR	52,840.21	0.03	1	1	1.4
W5PDY2	Uncharacterized protein	-	261,849.67	0	1	1	1.26
A0A452EBL1	Dihydrodiol dehydrogenase 3	LOC102177638	37,351.57	0.33	1	1	1.26
A0A452F4Q5	Dynein axonemal heavy chain 8	DNAH8	544,382.22	0	1	1	1.24
W5P1M4	Acetyl-CoA acyltransferase 2	ACAA2	42,086.45	0.41	1	3	1.24
W5NZV0	Heme oxygenase (biliverdin-producing)	HMOX2	43,155.73	0.3	1	1	1.22
W5PFZ8	Chromosome 20 C6orf141 homolog	C20H6orf141	20,592.16	0.05	1	1	1.21
A0A452G516	Oxysterol-binding protein	OSBPL11	85,188.23	0.02	1	1	1.21
W5NWX7	C-type lectin domain family 4 member A	CLEC4A	27,849.45	0.04	1	1	1.16
A0A452G154	NEDD1 gamma-tubulin ring complex targeting factor	NEDD1	71,754.15	0.01	1	1	1.14
W5PLM4	Aldo_ket_red domain-containing protein	-	32,650.38	0.33	1	1	1.1
W5PGI1	Protein kinase X-linked	PRKX	35,458.36	0.06	1	1	1.04
W5PTZ0	Membrane spanning 4-domains A5	MS4A5	22,614.45	0.07	1	1	1.02
A0A452DK46	Carbonic anhydrase	LOC102184245	29,121.58	0.41	1	12	1.01
A0A452G3K1	Actin related protein 10	ACTR10	44,622.44	0.02	1	1	0.99
A0A452EBG2	Tyrosine-protein kinase	ZAP70	69,121.32	0.03	1	1	0.99
A0A452EGG2	Rho GTPase activating protein 9	ARHGAP9	79,394.55	0.02	1	1	0.91
A0A452FP65	UDP-glucuronosyltransferase	LOC108633190	61,515.7	0.08	1	6	0.9
A0A452FEA5	Nicotinate-nucleotide pyrophosphorylase [carboxylating]	QPRT	30,980.17	0.02	1	1	0.88
A0A452FGG7	Sulfotransferase	SULT1C2	34,285.13	0.08	1	1	0.86
A0A452G0M2	Fraser extracellular matrix complex subunit 1	FRAS1	452,350.77	0	1	1	0.85
A0A452EWC8	Monocarboxylate transporter 1	SLC16A1	54,633.26	0.07	2	3	0.83
W5PLW4	Aldo_ket_red domain-containing protein	-	37,979.63	0.26	1	2	0.82
A0A452FG87	Microcephalin	MCPH1	84,188.78	0.01	1	1	0.82
W5QEK2	Tubulin tyrosine ligase like 4	TTLL4	133,873.12	0	1	1	0.81
A0A452ELR2	EvC ciliary complex subunit 2	EVC2	138,071.86	0.01	1	1	−0.81
W5PCE0	Phospholipase B-like	PLBD2	63,556.04	0.01	1	1	−0.81
P02080	Hemoglobin subunit beta-C(NA)	-	15,662.26	0.22	1	1	−0.81
W5PY97	Supervillin	SVIL	246,128.41	0.02	1	1	−0.81
A0A452G7Z1	Hematopoietic cell-specific Lyn substrate 1	HCLS1	52,381.17	0.02	1	1	−0.81
A0A452FBH2	2′-5′ oligoadenylate synthase	OAS1	41,810.87	0.13	1	2	−0.81
A0A452FBG8	Smoothelin	SMTN	98,772.31	0.09	6	7	−0.84
A0A452G7W8	Family with sequence similarity 110 member C	FAM110C	30,168.37	0.03	1	1	−0.84
W5P981	Non-specific serine/threonine protein kinase	AKT2	53,026.59	0.02	1	1	−0.84
A0A452E8I8	Uncharacterized protein	-	20,701.89	0.21	3	3	−0.84
W5Q1F0	ER lumen protein-retaining receptor	KDELR3	21,740.39	0.04	1	1	−0.84
A0A452F2B2	G protein subunit alpha o1	GNAO1	40,653.28	0.07	1	1	−0.84
A0A452DW39	60S ribosomal protein L29	-	16,612.21	0.08	1	4	−0.86
Q3LRQ1	Vitronectin	-	51,056.51	0.03	1	1	−0.86
W5PW78	Semaphorin 4C	SEMA4C	81,534.11	0.02	1	1	−0.86
A0A452ETB0	Collagen type XXVIII alpha 1 chain	COL28A1	115,775.7	0.01	1	2	−0.86
A0A452EHK0	Melanoma cell adhesion molecule	MCAM	71,399.1	0.1	6	9	−0.86
A0A452EFB7	Serine peptidase inhibitor, Kazal type 9	SPINK9	9851.55	0.29	2	3	−0.86
G1DFY5	Transforming growth factor beta-1-induced transcript 1 protein	TGFB1I1	50,629.24	0.02	1	1	−0.86
W5PMH6	Lipocalin 2	LCN2	23,169.06	0.09	1	1	−0.86
A0A452F2Y9	Actin alpha 2, smooth muscle	ACTA2	42,073.87	0.63	6	94	−0.89
A0A452E3D3	Perilipin 4	PLIN4	126,234.86	0.13	12	16	−0.89
A0A452E6P5	C-type lectin domain family 1 member B	CLEC1B	26,077.02	0.02	1	1	−0.89
A0A452ECP7	Carbonic anhydrase	CA3	29,707.9	0.25	5	8	−0.89
W5QHQ7	Nucleolin	NCL	73,334.54	0.15	1	1	−0.89
A0A452F4A0	Sorbin and SH3 domain containing 2	SORBS2	69,256.66	0.08	4	8	−0.92
W5P3N6	Beta-hexosaminidase	LOC101112162	62,702.01	0.08	1	1	−0.92
W5Q4S0	Collagen type III alpha 1 chain	COL3A1	139,701.25	0.08	8	35	−0.92
W5PFB6	CD177 molecule	CD177	47,924.93	0.03	1	1	−0.92
A0A452DUI8	Carcinoembryonic antigen-related cell adhesion molecule 7	LOC102172184	41,661.16	0.02	1	1	−0.94
A0A452FL86	TBC1 domain family member 32	TBC1D32	146,388.94	0.01	1	1	−0.94
W5P0I2	Complex I-B14.5a	NDUFA7	12,670.69	0.18	2	3	−0.94
A0A452FHU9	Collagen type I alpha 1 chain	COL1A1	139,952	0.18	15	179	−0.94
W5Q2A6	EF-hand domain family member B	EFHB	97,299.48	0.01	1	1	−0.94
W5PI04	TBC1 domain family member 13	TBC1D13	37,975.02	0.03	1	1	−0.94
A0A452FFP6	Beta-mannosidase	MANBA	102,118.69	0.03	1	1	−0.97
A0A452E5B5	Myosin heavy chain 11	MYH11	228,250.52	0.27	40	123	−0.97
W5NS84	Ret finger protein-like 4A	LOC101116932	32,111.24	0.03	1	1	−0.97
A0A452E7J1	Olfactory receptor	OR1G1	35,499.99	0.13	1	1	−1
A0A452EWP1	Synemin	SYNM	171,645.21	0.14	18	34	−1
A0A452EYA6	Protein kinase C	PRKCB	78,062.58	0.03	1	1	−1
W5PD82	Caldesmon 1	CALD1	89,883.93	0.14	9	20	−1.03
E7EC28	Thymosin beta	LOC102182562	5031.51	0.59	2	2	−1.03
A0A452DVI8	Anti-Muellerian hormone type-2 receptor	AMHR2	61,515.15	0.01	1	2	−1.06
A0A452EL11	Chondroitinsulfatase	GALNS	55,667.92	0.02	1	1	−1.06
A0A452EUR3	Guanylate cyclase	-	46,878.37	0.02	1	1	−1.06
A0A452E0C7	Olfactory receptor	-	35,416.77	0.05	1	1	−1.06
F8T866	MHC class II antigen	DQA	19,937.83	0.07	1	1	−1.09
A0A452FD11	Tropomyosin 1	TPM1	32,731.66	0.23	3	10	−1.09
A0A452F535	General transcription factor IIF subunit 2	-	28,635.95	0.06	1	1	−1.09
Q30DP7	Type II small proline-rich protein	SPRR2A	6489.94	0.3	1	4	−1.12
A0A452G1W6	Solute carrier family 9 member C2 (putative)	SLC9C2	129,059.02	0	1	2	−1.12
A0A224ATJ6	Olfactory receptor	OR4X2	29,701.85	0.1	1	1	−1.12
A0A452E4C4	ADAM metallopeptidase with thrombospondin type 1 motif 16	ADAMTS16	143,816.25	0	1	1	−1.15
A0A452G885	Keratin 4	KRT4	56,270.57	0.39	13	30	−1.15
A0A452EH97	Sorbin and SH3 domain containing 1	SORBS1	142,840.96	0.15	1	3	−1.18
W5PBX5	Mitogen-activated protein kinase kinase kinase 1	MAP3K1	157,095.44	0.01	1	1	−1.18
A1E458	Adipocyte-type fatty acid-binding protein	LOC100861279	14,805.48	0.32	3	9	−1.18
A0A452G893	S100 calcium binding protein A8	S100A8	10,399.34	0.16	2	3	−1.18
W5Q6L1	Keratin, type II microfibrillar, component 5-like	KRT85	40,944.41	0.05	1	1	−1.22
B5TQZ6	3beta-hydroxysteroid dehydrogenase/isomerase	3BHSD	43,108.07	0.07	1	1	−1.25
A0A452E9K4	Cardiac phospholamban	PLN	6229.38	0.21	1	1	−1.25
W5NV41	Ring finger protein 175	RNF175	38,416.48	0.07	1	1	−1.25
Q6S5L3	Major histocompatibility class II DQA1	Cahi-DQA1	28,131.18	0.09	2	2	−1.25
W5Q624	Keratin, type II cytoskeletal 73	KRT73	57,075.43	0.05	1	1	−1.29
A0A452EKJ0	Glycerophosphodiester phosphodiesterase domain containing 3	GDPD3	36,818.99	0.04	1	1	−1.32
A0A452EDZ4	Protein S100	S100A12	10,922.68	0.24	2	6	−1.32
R9WH56	MHC class I antigen	OLA-I	41,077.25	0.1	1	1	−1.36
Q9XSQ8	MAP28 protein	map28	18,000.42	0.08	1	1	−1.4
A0A452FL64	Resistin	RETN	12,192.84	0.24	1	1	−1.51
W5QB39	Glutamate receptor interacting protein 2	GRIP2	11,0290.8	0.01	1	1	−1.51
A0A068B4V9	Glutathione S-transferase	GSTA3	25,485.39	0.29	1	1	−2.32
A0A452F5B1	Semaphorin 3D	SEMA3D	90,605.53	0.04	1	1	−2.64

**Table 3:** The DEPs in the rumen epithelium of goat kids in the MCA group compared with the MRC group.

Protein_ID	UniProt Protein Name	Gene Symbol	Mass	Protein_Coverage	Uniq_Pep	Uniq_Spec	log2Fold Change
A0A452DTY3	Olfactory receptor	LOC102188949	35,991.4566	0.109	1	1	2.54
A0A452DVC9	Nanos C2HC-type zinc finger 3	NANOS3	19,372.3607	0.051	1	1	1.74
A0A452G516	Oxysterol-binding protein	OSBPL11	85,188.2306	0.017	1	1	1.46
A0A0H5FSL3	MHC class I antigen	Ovar-I	17,440.2913	0.17	1	1	1.20
W5QJ03	Dehydrogenase/reductase 7	DHRS7	38,509.2022	0.301	1	1	1.17
A0A452E7G7	Family with sequence similarity 71 member A	FAM71A	63,850.5707	0.024	1	1	1.14
A0A452FJ40	Membrane cofactor protein	LOC102169209	37,010.882	0.051	1	1	1.14
W5PFZ8	Chromosome 20 C6orf141 homolog	C20H6orf141	20,592.1564	0.049	1	1	1.14
A0A452G154	NEDD1 gamma-tubulin ring complex targeting factor	NEDD1	71,754.1493	0.012	1	1	1.10
A0A452FB93	CD276 molecule	CD276	57,663.7688	0.038	1	1	1.08
A0A452DUD2	DNA polymerase epsilon catalytic subunit	POLE	263,670.995	0.006	1	1	1.06
A0A452EWQ2	SPEM family member 2	SPEM2	56,745.5547	0.018	1	1	1.04
W5PRU6	Gap junction protein	GJA8	49,509.247	0.014	1	1	1.00
W5NWX7	C-type lectin domain-containing protein	CLEC4A	27,849.4487	0.042	1	1	0.97
A0A452EIF3	Transient receptor potential cation channel subfamily V member 4	TRPV4	98,757.5519	0.018	1	1	0.89
A0A452EB62	Uncharacterized protein	-	12,026.4189	0.09	2	2	−0.81
A0A452E2N5	Structural maintenance of chromosomes 6	SMC6	125,408.243	0.006	1	1	−0.81
A0A452E8Z1	Golgi associated kinase 1A	GASK1A	61,628.5216	0.011	1	1	−0.81
A0A452EZS9	RAB, member of RAS oncogene family like 3	RABL3	25,857.2573	0.026	1	1	−0.86
A0A452DKF9	4HBT domain-containing protein	-	13,848.0892	0.264	1	1	−0.86
A0A452FF33	Sodium/potassium-transporting ATPase subunit alpha	ATP1A3	108,265.03	0.1575	1	1	−0.86
A0A452EP10	Keratin, type II cytoskeletal 71	KRT71	55,027.6975	0.279	1	2	−0.86
W5PAY2	WW domain-containing protein	WWC2	129,564.039	0.005	1	1	−0.89
W5PMM4	Aldo_ket_red domain-containing protein	LOC106990122	37,134.3273	0.356	1	1	−0.89
W5Q6A5	Charged multivesicular body protein 2B	CHMP2B	23,990.3458	0.028	1	1	−0.92
W5Q9M6	WD_REPEATS_REGION domain-containing protein	-	47,001.9663	0.08	1	1	−0.94
G1DFX7	BolA-like protein 2	BOLA2	10,138.2385	0.081	1	1	−0.94
A0A452F7V6	Centrin 2	CETN2	19,100.5855	0.078	1	1	−1.00
E7EC28	Thymosin beta	LOC102182562	5031.50567	0.591	2	2	−1.09
G8FRI8	Copper chaperone of superoxide dismutase 1	-	23,909.9303	0.031	1	1	−1.09
W5QAL6	Formin like 3	FMNL3	117,941.038	0.012	1	1	−1.09
W5P9M9	SERPIN domain-containing protein	LOC101103862	44,569.5781	0.225	1	1	−1.18
A0A452F710	RNA 3′-terminal phosphate cyclase	RTCA	39,748.4576	0.044	1	1	−1.51
A0A452EYK5	Interferon induced protein with tetratricopeptide repeats 2	IFIT2	54,161.2524	0.013	1	1	−1.56
W5P8B8	Zinc finger protein 48	ZNF48	51,013.327	0.018	1	1	−1.60

## Data Availability

The related data in this study can be available by contacting to the corresponding author.

## Supplementary Materials

Supplementary materials can be found at https://www.mdpi.com/article/10.3390/biology12050684/s1, Table S1: Nutritional components of milk replacer, concentrate, and alfalfa pellets (dry matter basis) %; Table S2: iTRAQ labeling information; Table S3: Mascot Search Parameters; Table S4: Effect of solid feed supplantation on growth performance of goat kids; Table S5: Effects of solid feed supplantation on the rumen morphology in 20~60 days old of goat kids; Table S6: The identification of proteins in the study; Table S7: Gene ontology analysis of differently expressed proteins in the MRC vs. MRO group; Table S8: Gene ontology analysis of differently expressed proteins in the MCA vs. MRO group; Table S9: Gene ontology analysis of differently expressed proteins in the MCA vs. MRC group.
